# Phloroglucinol induces apoptosis via apoptotic signaling pathways in HT-29 colon cancer cells

**DOI:** 10.3892/or.2014.3355

**Published:** 2014-07-23

**Authors:** MI-HYE KANG, IN-HYE KIM, TAEK-JEO NG NAM

**Affiliations:** Department of Food and Life Science, Pukyong National University, Nam-gu, Busan 608-737, Republic of Korea

**Keywords:** apoptosis, HT-29 cells, phloroglucinol

## Abstract

Phloroglucinol is a polyphenolic compound that is used to treat and prevent several human diseases, as it exerts beneficial biological activities, including anti-oxidant, anti-inflammatory and anticancer properties. The aim of the present study was to investigate the effects of phloroglucinol on apoptotic signaling pathways in HT-29 colon cancer cells. The results indicated that phloroglucinol suppressed cell viability and induced apoptosis in HT-29 cells in a concentration-dependent manner. Phloroglucinol treatment of HT-29 cells resulted in characteristic apoptosis-related changes: altered Bcl-2 family proteins, cytochrome *c* release, and activation of caspase-3 and caspase-8. This study also showed that proteins involved in apoptosis were stimulated by treatment with phloroglucinol. These findings demonstrated that phloroglucinol exerts anticancer activity in HT-29 colon cancer cells through induction of apoptosis.

## Introduction

Colorectal carcinoma is a leading cause of cancer-related mortality worldwide (1). At present, radiotherapy or chemotherapy using cytotoxic drugs is the major method of cancer treatment, and many anticancer drugs have been applied clinically for colon cancer (2,3). However, due to increased concerns in regards to the side effects and drug resistance associated with current treatments, an ongoing search for natural antitumor therapies is underway (4,5). More effective drugs to treat colorectal carcinoma are necessary, and the development of natural therapeutics into potential anticancer agents is possible (6). In the past few decades, natural bioactive substances found in products were considered to be important antitumor drug sources. Marine seaweeds are widely used as a source of medicine and have attracted attention due to their minimal toxicity (7). Recent studies have demonstrated that the structural variety of phloroglucinol isolated from *Ecklonia cava* induces pharmacological activities by inhibiting apoptosis and protecting cells against oxidative stress. A number of studies showed that *Ecklonia cava* consists of many effective components (8). In particular, phloroglucinol is cytotoxic to breast cancer cells and has been studied to determine its pharmacological and immunological effects (9). However, the biological activities of phloroglucinol have yet to be fully elucidated. Thus, we selected phloroglucinol among many types of compounds, and evaluated the antitumor effects of phloroglucinol in colon cancer cells.

Apoptosis is the key to the normal growth and differentiation of diverse tissues. Thus, maintaining homeostasis in normal tissues requires a balance between cell proliferation and apoptosis. Apoptosis is a regulated and complex process leading to cell death characterized by nuclear fragmentation, chromatin condensation, membrane blebbing and cell shrinkage, which eliminates cancer cells without damaging surrounding tissues (10,11). Antitumorigenic agents induce apoptosis-related signaling in cancer cells while differentiation and development of cell tissues (12). Two major apoptosis signaling pathways caused by caspase activation fundamentally lead to apoptosis: an extrinsic pathway (death receptors) and an intrinsic pathway (mitochondrial) (13,14). Apoptosis regulated by the mitochondrial pathway, which involves molecules such as Bcl-2 family members, leads to the release of cytochrome *c* into the cytosol (15). The present study was performed to confirm that phloroglucinol inhibits the growth of HT-29 colon cancer cells and to determine the molecular mechanism of its anticancer effect by investigating apoptosis signaling pathways. Our findings suggest that phloroglucinol affects Fas-induced apoptosis, a potential factor in colon cancer prevention.

## Materials and methods

### Cell culture

Human colon cancer cells (ATCC HTB-38) and rat small intestine epithelial cells (IEC-6, ATCC CRL-1592) were purchased from the American Type Culture Collection (ATCC; Rockville, MD, USA). The cells were maintained in a humidified 5% CO_2_, 95% air, 37°C environment in Roswell Park Memorial Institute (RPMI)-1640 medium. Dulbecco’s modified Eagle’s medium (DMEM) was supplemented with penicillin/streptomycin (P/S), and HT-29 and IEC-6 cell cultures were supplemented with 10% fetal bovine serum (FBS; HyClone, Inc., South Logan, UT, USA). Cells were cultured to 80% confluency in 100-mm dishes. The medium was replaced every 2 days.

### Cell viability

Phloroglucinol was obtained from Sigma- Aldrich (St. Louis, MO, USA). The effects of diverse phloroglucinol concentrations on cellular proliferation of HT-29 and IEC-6 cells were examined colorimetrically after 24 h using the 3-(4,5-dimethylthiazol-2-yl)-5-(3-carboxymethoxy- phenyl)-2-(4-sulfophenyl)-2H-tetrazolium (MTS) assay with Cell Titer 96^®^ AQueous One solution reagent (Promega, Madison, WI, USA). To confirm viability, cells were seeded onto 96-well plates at 2×10^4^ cells/well in 100 μl medium and incubated for 24 h. Attached cells were maintained in serum-free medium (SFM) for 12 h, after which the medium was replaced with SFM containing phloroglucinol (0–50 μg/ml) for another 24 h. Cells were then incubated with MTS solution at 37°C for 30–60 min, and the absorbance of the solution in each well was measured at 490 nm using a microplate reader (Benchmark microplate reader; Bio-Rad Laboratories, Hercules, CA, USA).

### Caspase activity

Caspase activities were measured using caspase-3 substrate I (Ac-DEVD-pNA; 235400), caspase-8 substrate I (Ac-IETD-pNA), caspase-3 inhibitor [Z-D(OMe)- E-(Ome)-V-D(OMe)-FMK; 368057; Calbiochem, San Diego, CA, USA], and caspase-8 inhibitor (Z-IETD-FMK; R&D Systems, Minneapolis, MN, USA). HT-29 cells were seeded in culture dishes and grown to 60% confluency. These cells were treated with 50 μM caspase inhibitor for 1 h and phloroglucinol for 24 h, followed by addition of caspase lysis buffer [2.5 mM HEPES (pH 7.5) 5 mM EDTA, 2 mM DTT, 0.1% CHAPS]. A total of 100 μg/protein/100 μl was collected, and 2 μl of the substrate was added to the wells. Cells were incubated with caspase substrate in a shaking incubator at 37°C for 6 h. The absorbance at 405 nm was then determined using an enzyme-linked immunosorbent assay (ELISA) plate reader (Bio-Rad, Hercules, CA, USA).

### Apoptosis assay

Phloroglucinol treatment-induced apoptosis was determined using a Muse™ Annexin V and Dead Cell kit (EMD Millipore Co., Hayward, CA, USA). The cells were seeded onto 6-well plates at 60% confluency, and the medium was replaced with SFM for 4 h, followed by SFM containing phloroglucinol (0–50 μg/ml). After 24 h, the cells were collected in 1% FBS-RPMI-1640 medium and mixed using the Muse cell analyzer (EMD Millipore Co.).

### Western blotting

HT-29 cells in 100-mm dishes were cultured to 60% confluency and then incubated in SFM for 6 h, after which SFM containing phloroglucinol (0–50 μg/ml) was added to the cells for 24 h. To prepare whole-cell extracts, cells were washed with phosphate-buffered saline (PBS) and suspended in extraction buffer [20 mM Tris-HCl (pH 7.4), 150 mM NaCl, 1% NP-40, 1 mM EGTA, 1 mM EDTA, 0.25% Na-deoxycholate, 2.5 mM sodium pyrophosphate] containing protease inhibitors (1 mM sodium orthovanadate, 1 μg/ml aprotinin, 1 μg/ml pepstatin, 1 μg/ml leupeptin, 1 mM NaF, 1 mM PMSF) on ice. The extracts were centrifuged at 12,000 rpm for 10 min, and the supernatant was used for western blotting. Boiling sample buffer was added to the total cell lysate, and the samples were boiled for 10 min at 100°C. Proteins were separated in 7–15% SDS-PAGE and transferred onto polyvinylidene fluoride membranes (Millipore, Billerica, MA, USA). Membranes were blocked for 1 h 30 min at room temperature in blocking buffer [1% bovine serum albumin (BSA) in Tris-buffered saline-Tween-20 (TBS-T)], followed by incubation with primary antibodies (1:1,000 in 1% BSA/TBS-T) overnight at 4°C or for 2 h 30 min at room temperature. The membranes were then washed three times for 10 min in TBS-T, and horse-radish peroxidase (HRP)-conjugated goat, mouse or rabbit secondary antibody was added (1:10,000 in 1% BSA/TBS-T). Reactive bands were detected using Super Signal West Pico chemiluminescent substrate (Thermo Fisher Scientific Inc., Rockford, IL, USA) and expressed on Kodak X-ray film.

### Statistical analysis

Statistical analyses were performed using SPSS software (v. 18.0; SPSS Inc., Chicago, IL, USA). The results are represented as means ± standard deviation (SD). Differences between groups were determined using Duncan’s multiple range test. Statistical significance was set at P<0.05.

## Results

### Phloroglucinol inhibits proliferation of the HT-29 cells

**Figure 1:**
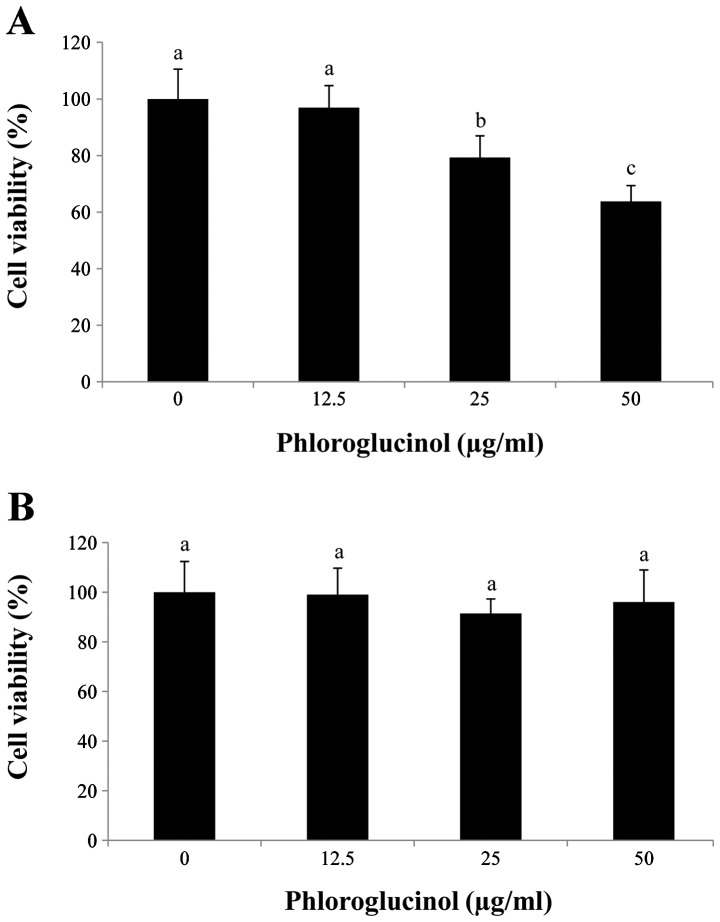
Effect of phloroglucinol on HT-29 colon cancer cell proliferation. (A) Effect of treatment with phloroglucinol (0, 12.5, 25 or 50 μg/ml) for 24 h on the growth inhibition in HT-29 cells. (B) Phloroglucinol-induced toxicity in intestinal epithelial cell-6 (IEC-6) cells.

In preliminary studies, we determined the effects of phloroglucinol treatment (0, 12.5, 25 and 50 μg/ml) on HT-29 colon cancer cells using the MTS assay (Fig. 1). Our data showed that phloroglucinol treatment decreased HT-29 cell proliferation in a concentration-dependent manner. In comparison with the control, 50 μg/ml phloroglucinol for 24 h inhibited cell viability by 60%. In contrast, IEC-6 cells were unaffected by the phloroglucinol treatment.

### Phloroglucinol induces morphological changes in the HT-29 cells

**Figure 2:**
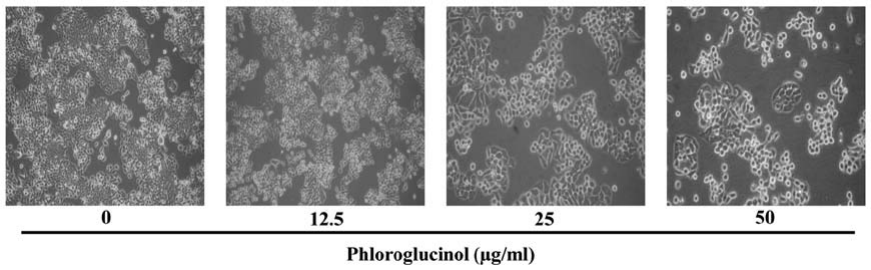
Morphological changes in HT-29 cells after treatment with phloroglucinol (0, 12.5, 25 or 50 μg/ml) for 24 h. Cells were observed using an optical microscope. Magnification, ×200.

Since phloroglucinol significantly reduced HT-29 cell viability, we used these concentrations to determine morphological changes using light microscopy (Fig. 2). We found that phloroglucinol decreased cell growth as well as cell size in a concentration-dependent manner.

### Phloroglucinol increases the rate of cellular apoptosis in a concentration-dependent manner as revealed by the Annexin V assay

**Figure 3:**
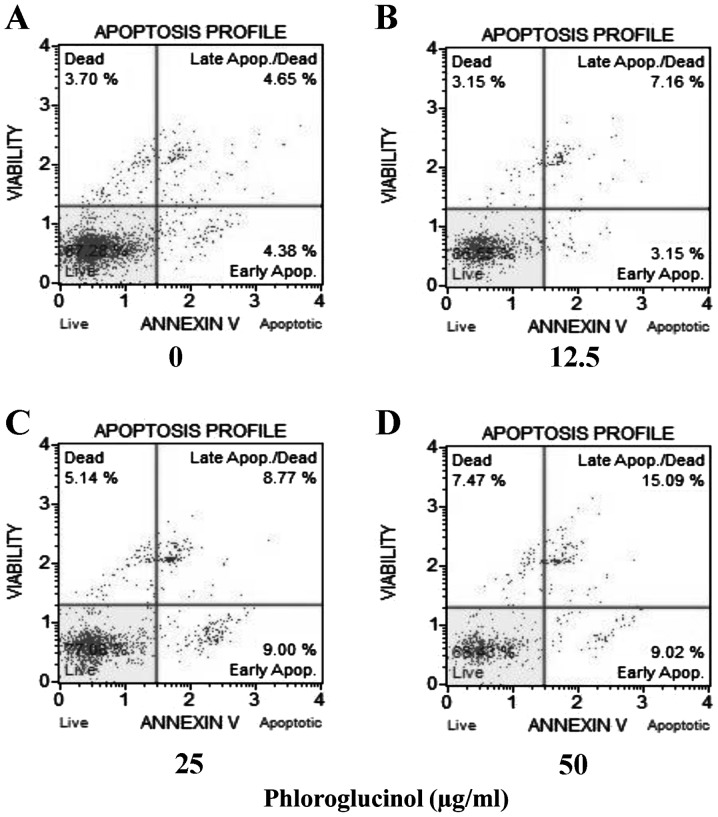
Phloroglucinol induces the apoptosis of HT-29 colon cancer cells. The percentages of apoptotic and necrotic cells were determined using an Annexin V and Dead Cell assay. Cells in the early stages of apoptosis showed Annexin V-positive and 7-AAD-negative staining, and cells in late apoptosis showed Annexin V-positive and 7-AAD-positive staining.

Colon cancer HT-29 cells were treated with phloroglucinol (0, 12.5, 25 and 50 μg/ml), and then the percentages of necrotic and apoptotic cells were evaluated by staining with 7-aminoactinomycin D (7-ADD) and Annexin V. In each analysis, non-apoptotic viable cells showed negative staining with Annexin V and 7-AAD. During early apoptosis, cells were Annexin V-positive and 7-AAD-negative; during late apoptosis, cells were Annexin V-positive and 7-AAD-positive. Mechanically injured cells were Annexin V-negative and 7-AAD-positive. In the present study, phloroglucinol treatment of HT-29 cells increased the percentage of apoptotic cells in a concentration-dependent manner. Untreated cell populations contained 87.28% living cells and 3.7% necrotic cells. Treatment with phloroglucinol (12.5, 25 or 50 μg/ml) for 24 h resulted in 10.31, 17.77 and 24.11% apoptotic cells, respectively (Fig. 3).

### Phloroglucinol induces the expression of apoptosis regulatory proteins

**Figure 4:**
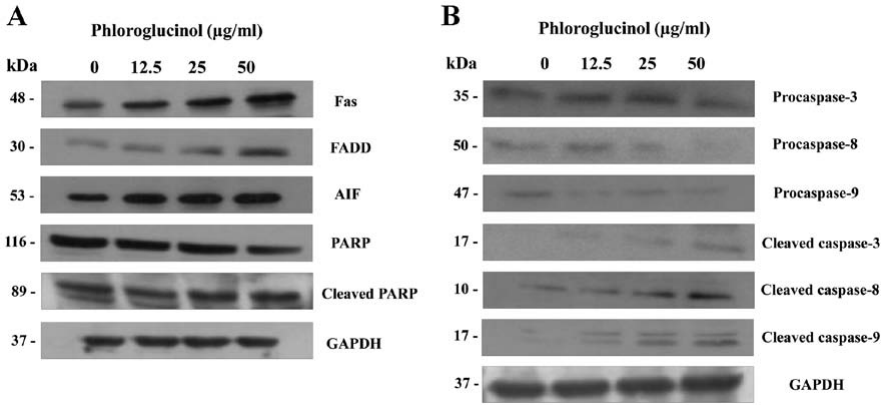
Phloroglucinol (0, 12.5, 25 and 50 μg/ml) alters the expression of apoptotic-related factors in HT-29 cells in a concentration-dependent manner. Phloroglucinol affects the expression of (A) apoptosis-related FAS, FADD, AIF, PARP proteins and (B) caspase proteins in HT-29 colon cancer cells. Phloroglucinol also induces DISC formation in HT-29 cells.

The multifactorial process of apoptosis is triggered by two main pathways (16). As shown in Fig. 4, we focused on the extrinsic pathway mediated by Fas. This pathway is regulated by activation of tumor necrosis factor (TNF) receptors and cell surface death receptors, such as Fas (CD95, APO-1) (17). The Fas signaling pathway upregulates downstream signal transduction, including members of the caspase family. Subsequently, apoptotic substrates are cleaved, including poly(ADP-ribose) polymerase (PARP) (18). In the present study, phloroglucinol treatment increased the expression of FAS and FADD and cleaved PARP, caspase-3, -8 and -9 (Fig. 4). These findings indicate that phloroglucinol induces apoptosis through the Fas signaling pathway.

### Phloroglucinol induces activation of caspase-3 and caspase-8

**Figure 5:**
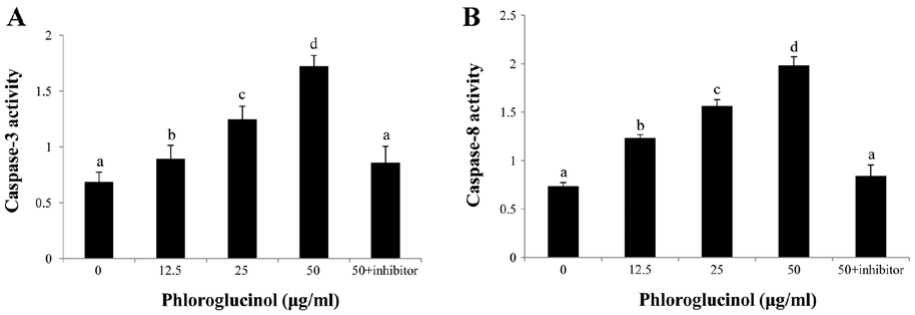
Concentration-dependent activation of (A) caspase-3 and (B) caspase-8 by phloroglucinol treatment (0, 12.5, 25 or 50 μg/ml) for 24 h in HT-29 cells. Caspase-3 and caspase-8 inhibitors were added at a concentration of 50 μg/ml.

To confirm which caspases are involved in phloroglucinol-induced apoptosis, caspase inhibitors were evaluated. Our results demonstrated that caspase-3 and -8 inhibitors potently repressed phloroglucinol-induced apoptosis (Fig. 5). These results indicate that caspase-3 and caspase-8 activation are involved in phloroglucinol-induced apoptosis.

### Phloroglucinol induces mitochondrial membrane potential dissipation and cytochrome c release

**Figure 6:**
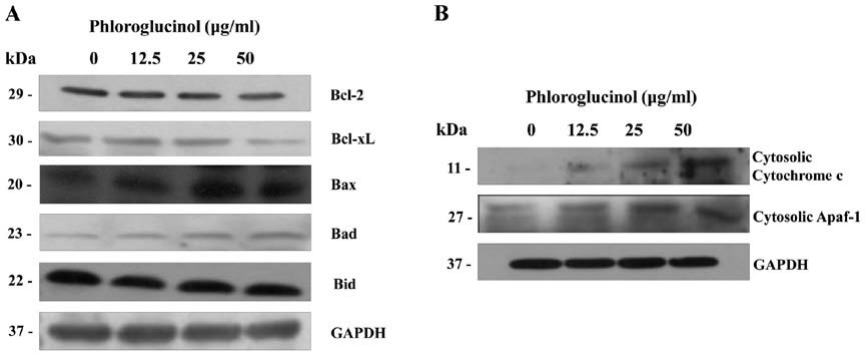
(A) Effect of 24-h phloroglucinol treatment on the expression levels of anti-apoptotic Bcl-2 and Bcl-xL and pro-apoptotic Bax and Bad proteins in HT-29 cells. (B) Effects of phloroglucinol on cytosolic cytochrome *c* and apoptotic protease activating factor-1 (Apaf-1) levels in HT-29 cells. Phloroglucinol increased the cytosolic expression of cytochrome *c* and Apaf-1.

The mitochondria- related pathway is a significant apoptotic pathway characterized by cytochrome *c* release from the mitochondria into the cytosol and by disorganized mitochondrial transmembrane potential (19). In addition, the mitochondrial apoptosis pathway commonly involves Bcl-2 family proteins. Variations in the proportion of these members affect the life or death of cells (20). In order to determine whether cytochrome *c* release is enhanced in phloroglucinol-treated HT-29 cells, we analyzed the cytosolic expression levels of cytochrome *c* and Bcl-2 family proteins. Levels of Bcl-xL and Bcl-2 expression were simultaneously suppressed (Fig. 6A); conversely, levels of Bax, Bad, and Bid expression were increased. Subsequently, the expression levels of cytochrome c and apoptotic protease activating factor-1 (Apaf-1) in the cytosol were also increased (Fig. 6B). These results indicate that collaboration among the Bcl-2 family proteins was involved in mitochondrial change during the advancement of apoptosis by phloroglucinol.

## Discussion

Apoptosis is an essential process involved in homeostasis and maintenance of multicellular organisms by extirpating superfluous cells (21). Furthermore, induction of apoptosis is critical to remedy cancer (22). In this study, we investigated the interaction between the extrinsic and intrinsic pathways of apoptosis induced by phloroglucinol in human colon cancer HT-29 cells. We determined that phloroglucinol inhibits cell proliferation by inducing apoptosis: we observed altered cellular morphology (whole cell numbers and cell size were reduced in a density-dependent manner) following changes in the levels of pro-apoptotic and anti-apoptotic proteins and caspase activation. Moreover, we also showed evidence that the cell apoptosis ratio increased following treatment with high phloroglucinol concentrations.

The death receptor-mediated pathway is triggered by FasL and its receptor FADD (23,24). FADD then induces caspase-8 via interaction with death effector regions, leading to activation of caspase-8 and the apoptotic protease cascade via effector caspase activation (25,26). In this study, phloroglucinol increased the extrinsic signaling protein expression levels of Fas, FADD and caspase-8.

Caspases, which are cysteine proteases, play an important role in regulating the apoptotic response (27). Activation of apoptotic signaling pathways results in cytochrome *c* release from the mitochondria in concert with cleavage and activation of caspase-9, which in turn also induces caspase-3 activation (28,29). In the present study, we found that phloroglucinol- induced HT-29 cell apoptosis was associated with activation of caspase-3, -8 and -9.

Moreover, cell apoptosis is modulated by Bcl-2 family members, which negatively or positively regulate apoptosis. Anti-apoptotic Bcl-2 and Bcl-xL proteins are able to intercept diverse apoptotic signals, whereas pro-apoptotic proteins, such as Bax and Bad, can cause the release of apoptosis palpating factors into the cytoplasm (30,31). Our data showed that phloroglucinol induced a decrease in Bcl-2 and Bcl-xL expression and an increase in Bax and Bad expression. This alteration may trigger the release of cytochrome *c* and Apaf-1 into the cytosol and the cleavage of poly(ADP-ribose) polymerase (32). In this study, the release of apoptosis-promoting factors such as cytosolic cytochrome *c* and Apaf-1 was increased following phloroglucinol treatment. Therefore, these findings suggest that phloroglucinol induces apoptosis through mitochondrial dysfunction by modulating the protein levels of Bcl-2 family members. These findings may aid in the understanding of the mechanism underlying induction of apoptosis by phloroglucinol in carcinoma cells.

In conclusion, our study demonstrated that phloroglucinol significantly inhibited growth and induced apoptosis of HT-29 colon cancer cells via both the extrinsic and intrinsic cell death signaling pathways. This research provides a mechanism for the antitumorigenic activity of phloroglucinol. In the apoptotic process, phloroglucinol upregulated pro-apoptotic and downregulated anti-apoptotic proteins, followed by caspase activation. Our results partially explain the effect of phloroglucinol on HT-29 colon cancer cell apoptosis. Although a fully detailed mechanism is not clear, phloroglucinol could be used as a potential therapeutic candidate in the prevention or treatment of colorectal cancer in the future.
